# Telehealth in community mental health centers during the COVID-19 pandemic in Peru: A qualitative study with key stakeholders

**DOI:** 10.1016/j.ssmmh.2023.100287

**Published:** 2024-06

**Authors:** Rubí Paredes-Angeles, Victoria Cavero, Ana L. Vilela-Estrada, Noelia Cusihuaman-Lope, David Villarreal-Zegarra, Francisco Diez-Canseco

**Affiliations:** a CRONICAS Center of Excellence in Chronic Diseases, Universidad Peruana Cayetano Heredia, Lima, Peru; b Instituto Peruano de Orientación Psicológica, Lima, Peru

**Keywords:** Telehealth, Mental health, COVID-19, Key stakeholders, Peru

## Abstract

**Aim:**

To describe the perceptions and experiences of key stakeholders to understand the use of telehealth in community mental health centers (CMHCs) during the COVID-19 pandemic in Lima and Callao, Peru.

**Methods:**

A qualitative study was carried out in four CMHCs in Lima and Callao, Peru. Forty-nine individual semi-structured interviews were conducted between September 2021 and March 2022, considering CMHCs' users and their relatives, health and administrative workers, directors, as well as local and national policymakers. Data was analyzed using thematic analysis.

**Results:**

Regarding the transition from in-person care to telehealth, CMHCs' directors and workers identified some of the regulations issued by the Government during the pandemic, such as the continuity of care through telehealth, especially for pregnant women and for people with comorbidities related to COVID-19. Regarding benefits, workers and users indicated that it allowed better communication, such as constant follow-ups. Directors and workers recognized that Google Drive facilitated access to user information, since they did not have an electronic medical record. Additionally, workers said they used social media to share educational information on mental health, and explained that some new users began their treatment this way. Regarding difficulties, participants reported a lack of devices and poor internet connection in CMHCs. Users mentioned that scheduling an appointment was difficult because the phone lines were usually saturated, and they could not find available appointments.

**Conclusion:**

Although the pandemic forced an immediate and disruptive change towards telehealth, CMHCs were able to adapt most of their services. This study reports the adaptations made by CMHCs to move from in-person to remote care, identifying the benefits and challenges faced, information that can be used for the nationwide implementation of telehealth in CMHCs. We recommend ensuring technological equipment and internet connection and adapt the telehealth system to make it responsive to the routine practices of CMHCs.

## 1 Background

In 2020, the COVID-19 pandemic and the subsequent measures taken by governments to contain its expansion, such as mandatory social isolation and closure of public spaces (Calderón Peralvo et al., 2022), caused a severe psychosocial impact on a global scale (Rajkumar et al., 2022). Several governments, at the beginning of 2020, also restricted the use of health services, especially those considered non-essential to treat COVID-19, such as mental health services (Duden et al., 2022). Since mental health treatments were interrupted during the beginning of the pandemic, symptoms worsened in people with mental disorders, especially for those living in low- and middle-income countries (LMICs) (WHO, 2022).

To ensure the continuity of care in mental health services, several governments used telehealth (Frank et al., 2021). Telehealth is a distance health service provided by health personnel that includes 4 axes: provision of health services (i.e. telemedicine), management of health services, health education for the general population and strengthening the capacities of health workers (Gobperu, 2023). According to a World Health Organization (WHO) report, more than 80% of high-income countries (HICs) but less than 50% of LMICs used telemedicine to deliver mental health care; a difference that may be related to the scarce resources allocated to mental health services in LMICs (WHO, 2022). To date, most of the evidence showing the efficacy of technology-based mental health services during the pandemic comes from HICs (Pierce et al., 2020). In contrast, in LMICs, a lack of well-equipped telehealth services, with limited access to electricity, devices, or internet were reported during the pandemic (Kola et al., 2021).

Peru, a middle-income country, was among those nations providing remote care during the pandemic. In the COVID-19 context, the first regulation that the Peruvian Government approved for health centers to migrate to remote care was published in March 2020 (Peruvian Ministry of Health, 2020a), while the normative to incorporate telehealth for mental health care was published in April 1st, 2020, less than a month after the first case of COVID-19 was reported in Peru, in March 6th, 2020 (Peruvian Ministry of Health, 2020b). The telehealth services proposed were mainly for the provision of remote consultations, scheduling appointments, and electronic prescriptions (Peruvian Ministry of Health, 2020b).

During 2020, more policies regarding the use of telehealth for mental health care were published (Peruvian Government, 2023; Peruvian Ministry of Health, 2020c). Some emphasized the use of telehealth in the 249 community mental health centers (CMHCs) of the country (Peruvian Government, 2023), which are facilities that provide outpatient and specialized care to people with mental disorders. CMHCs were created in 2015 (Andina, 2015) by the Peruvian Government with a community-based model of care, offering services such as psychiatry, psychology, family medicine, nursing, pharmacy, social work, among others (Peruvian Ministry of Health, 2017). These facilities served the largest number of mental health services' users in Peru during the pandemic (RPP Group, 2021). Peruvian policies on telehealth indicated the allocation of technological infrastructure (i.e. internet, desktops, printers), development of telehealth platforms and electronic medical records, and training of the health personnel to provide remote care (Peruvian Ministry of Health, 2020c). However, the implementation of telehealth to provide mental health care in CMHCs, its benefits, and difficulties, has not been studied. This qualitative study aims to describe the use of telehealth in CMHCs during the COVID-19 pandemic in Lima and Callao, Peru, identifying its perceived benefits and challenges, from the perspective of different key stakeholders (i.e. CMHCs users and their relatives, health and administrative workers, directors, local and national policymakers) (Patil et al., 2021).

## 2 Methods

### 2.1 Setting

This study was conducted in three CMHCs based in different areas of Lima, capital of Peru (North, South, and Center), and one located in Callao, a province adjacent to Lima. Lima and Callao represent 30% approximately of the Peruvian population (National Institute of Statistics and Informatics, 2022), and have 53 out of the 249 CMHCs (21%) of Peru (Peruvian Ministry of Health, 2020a).

The CMHCs were created with a community-based care model in order to focus on people, family and communities, encouraging their effective participation from the planning to the evaluation of the processes implemented for the promotion and protection of their mental health. CMHCs have three functions. First, CMHCs are primary care centers that provide specialized mental health care to people with common but primarily severe mental disorders, including family medicine, psychiatry, psychology, nursing, pharmacy, language therapy, and social work services (Peruvian Ministry of Health, 2020b). Second, CMHCs articulate actions with the social actors of the community, which involves the creation and activation of the social and community networks for the promotion of mental health, and conduct preventative and promotional mental health activities, such as in schools, shelters, or public spaces (Peruvian Ministry of Health, 2020b). Finally, CMHCs are also supposed to train and supervise the personnel of primary health care services in the detection and treatment of common mental disorders, and the referral of the most complex cases to secondary or tertiary care levels (Peruvian Ministry of Health, 2020b). Before the COVID-19 pandemic, these functions were conducted in person (Peruvian Ministry of Health, 2020b). Each of the CMHCs included in this study corresponded to a different Health Direction (Northern Lima, Southern Lima, Center Lima, and Callao), which were in charge of supervising the care provided in healthcare facilities. Similarly, these Health Directions are supervised by the Ministry of Health (MoH), which has a specific Mental Health Direction in charge of all mental health regulations nationwide, including the ones implemented in the CMHCs.

### 2.2 Study design

A qualitative phenomenological study was conducted (Creswell and Creswell, 2018) to describe the lived experiences of key stakeholders involved in the provision of telehealth for mental health care at CMHCs of Lima and Callao, Peru, during the COVID-19 pandemic.

### 2.3 Participants

A total of 49 participants were included and most of them (75.5%) were female. All participants were selected using a stratified purposive sampling (Patton, 1990), resulting in seven profiles: i. CMHCs users (n = 16) ii. Users' relatives (n = 8), iii. CMHCs health workers (n = 8), iv. CMHCs administrative workers (n = 8), v. CMHCs directors (n = 4), vi. Local policymakers (n = 3), and vii. National policymakers (n = 2). All participants were 18 or older. The specific inclusion criteria for each profile were as follows:

#### 2.3.1 CMHCs users

Two regular and two new users of each CMHC were included. Regular users were those who used to receive care in the CMHC before March 15th, 2020, which was operationalized as having received care in the CMHC at least three times during the previous three months. New users were those who began their care at the CMHC after March 15th, 2020, and had at least three consultations before our interview. We selected this date because it was when the state of emergency began in Peru and health services moved to remote care. In terms of diagnosis, users included were also divided into those who had a severe mental disorder (i.e. schizophrenia, severe depression) and a common mental disorder (anxiety, mild depression). Those unable to voluntarily decide their participation in an interview were excluded (e.g. users in an acute stage of their symptoms).

#### 2.3.2 Relatives of CMHCs' users

One relative of a regular user and one of a new user per CMHC were included. All relatives had to be knowledgeable about the care that the user received in the CMHC during the COVID-19 pandemic, and also before the pandemic for regular users.

#### 2.3.3 CMHCs health workers

Two health workers of each CMHC were included. They had to be working in the CMHC for at least three months before March 15, 2020, and provide clinical care to CMHC’s users.

#### 2.3.4 CMHCs administrative workers

Two administrative workers for each CMHC were included. They had to be working in the CMHC for at least three months before March 15, 2020, and perform non-clinical activities, such as scheduling appointments, storing paper-based health records, or managing human resources.

#### 2.3.5 CMHCs directors

The current directors of the four selected CMHCs were interviewed. They were usually a medical doctor or a psychologist in charge of managing the whole CMCH and with direct communication with its local Health Direction.

#### 2.3.6 Local policymakers

For mental health, Health Directions have a mental health coordinator, whose duties include planning and monitoring the care provided at the CMHCs. Three mental health coordinators were interviewed, since one of them was hard to reach.

#### 2.3.7 National policymakers

Two policymakers working at the Mental Health Directorate of the MoH were interviewed. To participate, they had to be informed about the care provided at the CMHCs during the COVID-19 pandemic.

### 2.4 Data collection and procedures

All data was collected using semi-structured interviews. Specific interview guides were designed for each of the seven participant’s profiles and areas of exploration in the interview guides are shown in Table 1. To enter the CMHCs, we reached the directors of the CMHCs to request their authorization and invite them to participate. Additionally, we asked them to put us in contact with key CMCHs' informants (e.g. nurses) to help us recruit users, relatives and workers from the CMHC. We did not approach users directly because the CMHC could not provide us with their contact information since they were a vulnerable population. Thereby, the key CMHC informant helped us to identify the users who met our inclusion criteria, and then reached them to explain the study and invite them to participate. If they were interested in participating, the key CMHC informant gave us their contact information. For national policymakers' recruitment, we reached them directly since the research team has already worked with them in the past. Finally, national policymakers gave us the contact information of local policymakers so we could reach them. All potential participants were reached by phone to invite them to participate. They received a copy of the informed consent form by email or WhatsApp, and were informed about the study purpose, procedures, potential risks and benefits, among others. If they agreed to participate, they send the research team a signed informed consent and scheduled the interview.

**Table 1:** Areas of exploration in the interview guides across profiles.

Profile	Areas of exploration in the interview guides
CMHCs users	- Experiences with the care provided at the CMHCs during the COVID-19 pandemic (telehealth content was explored) - Perceived barriers and facilitators to continue the care and services received during the pandemic - Changes in the care received due to the pandemic (for regular users only)
Relatives of CMHCs' users	- Experiences with the care provided at the CMHCs during the COVID-19 pandemic (telehealth content was explored) - Perceived barriers and facilitators to continue the care and services received by their relative - Perceived impact of the COVID-19 pandemic on the mental health of their relative - Changes in the care received by their relative due to the pandemic (for relatives of regular users only)
CMHCs health, administrative workers and directors	- Experiences with the care provided at the CMHCs during the COVID-19 pandemic (telehealth content was explored) - Perceived barriers and facilitators to continue the care and services provided during the pandemic - Changes in the care provided due to the pandemic - Perceived impact of the pandemic on workers' mental health and available support for health workers
Local and national policymakers	- Planned and unplanned changes in the care provided in the CMHC due to the pandemic - Perceived barriers and facilitators to continue the care and services provided during the pandemic - Coordination of the CMHCs with local networks - Training for CMHCs workers - Human resources and infrastructure implemented to continue the care provided at CMHCs - Use of technology for telemedicine care in CMHCs

Interviews were conducted remotely (by video call or phone) to avoid exposure to COVID-19, by three members of the research team with extensive experience conducting qualitative interviews with different stakeholders. Interviews lasted 69 minutes on average for policymakers, CMHC directors, and workers; and 32 minutes for users and their relatives. All interviews were conducted between September 2021 and March 2022.

### 2.5 Data analysis

Data was analyzed using thematic analysis (Braun and Clarke, 2008), by three members of the research team trained to analyze qualitative data. First, an initial codebook was developed, which was then reviewed with senior members of the research team to refine the terms and definitions (Strauss and Corbin, 1990). Second, four interviews were jointly reviewed and coded by the three members to standardize the coding process. Then, a fifth interview was coded separately by the three members and their coding were compared. Once they achieved more than 95% match in their coding (Saldana, 2016), the remaining interviews were divided among them, according to the participant profiles. The most experienced researchers analyzed data from local and national policymakers since they had more experience and knowledge about public policies on mental health and health systems' dynamics. Several joint coding and analysis sessions were conducted by the research team to ensure high-quality coding.

### 2.6 Ethics

This study received ethical approval from the Institutional Ethics Committee of Universidad Peruana Cayetano Heredia, in Lima, Peru (Approval record 573-33-20). Participation was voluntary, and all the informants provided their informed consent form before the interview. All research team members completed ethical training in good clinical practices and human participants' research.

## 3 Results

The study findings are organized in three main topics: i. Transition from in-person care to telehealth in CMHCs, ii. Perceived benefits of using telehealth, and iii. Difficulties in the provision of mental health care through telehealth.

### 3.1 Transition to telehealth

#### 3.1.1 Regulations

Many CMHCs' workers and directors said that, at the beginning of the pandemic, they were informed by their Health Directions that the MoH enacted some regulations indicating that CMHCs had to use telehealth. According to all the CMHCs' workers, the normative indicated that the number of workers attending the CMHC should be reduced -prioritizing those with chronic diseases (i.e. diabetes, hypertension) and pregnant women-, and that most activities should be conducted remotely. These CMHCs' workers said that they returned to in-person activities several months later, when COVID-19 cases decreased.

*My colleagues went to the CMHC in person, but in my case I couldn’t (go) because of my hypertension diagnosis, so I continued with remote work. Later, a regulation came out again that explained that people with controlled hypertension also had to go in person … So, I rejoined the CMHC (Health worker, CMHC2)*

In that context, according to some health workers, the CMHCs' users were informed, through phone calls or social networks, that they should avoid attending the CMHC. Only those needing medication or experiencing a psychiatric emergency would be received. They also said that most CMHCs' activities, such as the scheduling of appointments, follow-ups, clinical consultations, home visits, etc., were carried out by phone, using WhatsApp or video calls (i.e. Zoom).

*The Directorate of Mental Health issued a statement, in which they told us that our work will be done remotely, providing mental health support for those affected by the pandemic and continuing our care for previous users (Administrative worker, CMHC3).*

Finally, CMHCs' workers and directors mentioned that their Health Directions prioritized some services to provide in-person care at the beginning of the pandemic: medicine (psychiatry, family medicine), nursing, pharmacy, and admission. Some nurses and doctors expressed that they disagreed with this decision, arguing that this placed them at a higher risk of acquiring COVID-19, specially because they did not have the “appropriate protective equipment” to prevent an infection. Most nurses suggested that members from all services should attend in person and rotate, since they considered unfair that psychologists or physiotherapists were exempt from in-person activities. They said that this recommendation was not implemented initially, but after some months, when several workers were infected with COVID-19, CMHCs required that all professionals should work in-person, but in different days and shifts, to avoid overcrowding the CMHCs.

#### 3.1.2 Changes in CMHCs services

The services offered by the CMHCs were: individual care, group activities, home visits, dispensing of medicines, and clinical support for primary care workers from other health centers.

For **individual care**, all workers and directors mentioned that their CMHCs adapted their services to provide remote care using different technological tools. For instance, CMHCs' workers expressed that they tried to use Zoom or Google Meet to provide clinical care at the beginning of the pandemic; however, they said that users were not able to use them as expected, because of their poor internet connection and lack of devices (i.e. cellphone, laptop). Some workers and directors also reported that users did not know how to use Zoom, Meet or WhatsApp, so they had to learn users how to enter to a video call or download apps, particularly adults and older adults, and many health workers and policymakers said that some users did not have smartphones, only basic cell phones. For these reasons, workers and directors gave users the option to have the appointments by phone calls, an alternative that was very well received, from the workers' perspective. Users also expressed that they preferred these calls because “everyone has a phone”, and because they were called frequently by the CMHCs' personnel. Thereby, workers emphasized that phone calls became the most frequent and preferred modality of care delivery, over Zoom and Google Meet.

Most CMHCs' directors, health and administrative workers mentioned that they used Google Drive for several activities during the pandemic, such as healthcare records, because they did not have an electronic medical record system. Most health workers revealed they used Spreadsheets (Excel) to complete their mandatory forms remotely to document and share their consultations, so, if a psychiatrist wanted to see the care provided to the users in the psychology service, they could access the psychology Drive folder and view them and some of them said that also used it to schedule their appointments and count the number of users attended daily and monthly.

According to some CMCHs' health workers and directors, two telehealth platforms were used to provide remote mental health care during the pandemic. According to directors, one was a web platform, called MentalCom, which was specifically created by the Peruvian National Institute of Mental Health to manage appointments, record clinical data, and produce reports in real time in the CMHCs. However, only administrative workers from one of the four CMHCs reported having used it. One of them mentioned they received a 3-month training by their Health Direction and, after using MentalCom, she considered it a useful tool because “it had all the functions that administrative workers needed”.

When the interviews were conducted, informants mentioned that MentalCom was no longer being used in the CMHCs because they did not have the equipment to use the platform, such as desktops and a stable internet connection. Administrative workers from other two CMHCs mentioned that they used e-QHALI, an electronic medicalrecord designed by the MoH for primary care centers that included a mental health module. These administrative workers stated that e-QHALI was only used to schedule appointments at the CMHCs because they said that they needed other devices, such as desktops and printers, to use other features of the software. However, administrative workers commented that they considered e-QHALI an effective and easy-to-use tool. Workers explained that, even though both Mentalcom and e-QHALI were created to be used by health and administrative workers, finally only some administrative workers used it due to a lack of technological resources. In contrast, a local policymaker stated that, only at the beginning of the pandemic, the use of the platforms was complicated, but afterwards, the workers used the platform’s features without difficulties.

Regarding **group activities,** commonly run by the CMHCs before the COVID-19, both CHMCs' users and workers reported that they were canceled at the beginning of the pandemic. According to the workers, some months later, all CMHCs tried to resume their group activities remotely, but this was difficult due to the poor internet connection of workers and users; therefore, three CMHCs canceled them and only one continued. The CMHC’s workers that continued providing group activities commented that they performed the remote activities without video, to reduce bandwidth usage, or use their own cell phones' internet data. Workers from the other three CMHCs said that almost 18 months after the onset of the pandemic, these activities were resumed in person at most CMHC’s, implementing biosecurity measures to avoid contagion (e.g., social distance, use of masks, hand-washing).

*At the beginning of the pandemic, group activities were totally restricted. We did not allow many users to enter the CMHC. Group activities were resumed only after the second wave. They resumed in the middle of this year … they resumed with, with a small (group), six, seven users in a … in a spacious place, right? (CMHC director, CMHC4).*

A director mentioned that the physical education workshops provided for users of CMHCs and the psychosocial club for individuals with severe mental disorders (e.g. schizophrenia) were discontinued, and another director explained that for the resumption of group activities, priority was given to users who were victims of domestic violence and those with severe mental disorders.

The CMHCs' workers and directors mentioned that, in the pandemic, they prioritized the **dispensing of medicines** to allow users to continue their treatment and collect their medicines rapidly. To do so, workers commented that nurses or pharmacists called the users to coordinate a date and time to collect their medication, avoiding cues or overcrowding. Users and health workers talked about these follow-up calls to monitor if the medication was being used as indicated. Users expressed that they liked these calls since they felt cared for and protected.

*The calls make you feel more important, that they care about you, right? At no time, as I tell you, did they faint. They did not take the pandemic as an excuse to leave the user, in no way. On the contrary, they were more thoughtful than ever (Regular user, CMHC2).*

Adittionally, policymakers mentioned that when users did not want to attend to the CMHC to pick up their medication, either due to difficulty in transportation or fear of contagion, healthcare workers delivered it to their homes after coordinating with the user or their family through a phone call. A national policymaker mentioned that this was done because one of the policies of the Peruvian government established that ensuring the continuity of medication in mental health was necessary.

According to different informants, some CMHCs were able to replace **home visits** with video calls. Social workers and nurses commented that they used video calls with users and their family members, asking them to walk their homes to show the user’s bedroom, or talk about how they were organizing the medication and how the user felt with the treatment. Directors and policymakers explained that only in case of an emergency and with some users with severe mental disorders, health workers visited the users' homes, using protective personal equipment.

*… In some cases, of severe mental disorders, workers had to visit users, but the workers stayed at the door of the house, right? Outside, on the sidewalk, taking those distances because it was necessary, in some cases, to make visits (Local policymaker).*

Regarding **clinical** **support** **for health workers from other health centers**, some CMHCs' health workers mentioned that during pandemic they continued this training remotely, using Zoom or Google Meets. According to workers, this support involves advising other healthcare workers on the management of patients with mild mental health problems, and if the workers at the CMHCs detect that a case requires more specialized management, they recommend that the staff of these other centers refer them to the CMHCs. Additionally, some health workers reported that an activity that arose during the pandemic was the provision of remote psychological support to primary care personnel, who were “emotionally affected” by the loss of their relatives or presenting symptoms of anxiety or depression, so they were incorporated as new users of the CMHCs. Two workers said that they prioritized the health personnel who needed mental health care, so their pathway of care was faster.

However, many CMHCs' health workers and directors commented that training and support activities were exhausting for them so they felt that they commonly cared about others' mental health but neglected their own wellbeing. National policymakers reflected on this, saying that even though the current mental health policies had guidelines to attend the mental health of health workers in general, those in charge of providing such care were the CMHCs workers, and this specific group did not have any specific guideline to support their mental health.

### 3.2 Perceived benefits of using telehealth

One of the main benefits, highlighted by informants from all profiles, was that technology allowed the continuity of care during the pandemic. Health workers emphasized that telehealth enabled them to attend users who were at home, those who lived far from the CMHC, or even those users that moved to other cities during the pandemic but previously received treatment in the CMHC. Users and their relatives appreciated that they did not need to attend the CMHC in person, because they were afraid of being infected with COVID-19. In fact, when most activities returned to be in-person, some users and relatives expressed that they did not attend because of this fear.

*At the beginning of the pandemic, I was very … very worried, if I went out with my relative, we would risk getting infected … I was afraid, I resisted going out … I indeed preferred that the appointments were remote (Relative of regular user, CMHC1).*

From the CMHCs' workers' and directors' point of view, the use of technology allowed better communication with users, especially to confirm appointments and conduct follow-ups. Users and relatives also mentioned this as a benefit, since they liked that CMHCs' workers called them more frequently than before the pandemic.

*Yes, the CMHC’s workers called me more often, to ask me how I was doing, how I was, about my emotions … in the case of the psychologist. The doctor called me every time she had to medicate me and also to know how I was. The truth is that during the pandemic they have been more thoughtful with us, or at least I felt that way (Regular user, CMHC2).*

Another benefit mentioned by CMHCs' workers was the use of Spreadsheets in Google Drive to facilitate that all providers could access to users' information (e.g., diagnosis, current treatment, appointment dates) and other services' information.

A benefit, highlighted by social workers, was that technology eased their coordination with other institutions, such as the police, women’s emergency centers, shelters, and schools. Social workers pointed out that before the pandemic, this communication was only face-to-face and thereby very scarce, however, during the pandemic, they were able to do it by phone or email, more frequently and rapidly. As a consequence, the social workers recommended the continuation of these methods to assure such coordination.

*Before [the pandemic] a user derived from another institution came with the document (reference) to the CMHC, in person, to leave it here and receive care. Now it has naturally changed, right? [Now] it is from institution to institution, by email (Health worker, CMHC4).*

Workers and directors expressed that using technology also made it easier to have meetings with their coworkers, for example, Zoom or Google Meet were used to coordinate or make adjustments to the care provided at the CMHC. They also said that learning how to use these platforms was an additional benefit. Before the pandemic, they commented that they mainly had in person meetings in the CMHC and finding a time was sometimes challenging. However, with the virtual meetings, they said that they had more flexibility to arrange a conversation at different times and connect from different places.

Finally, some health workers mentioned that social media made it possible to provide health and mental health educational information to the general population (e.g., key concepts about anxiety, stress, and COVID-19 symptoms). Many health workers said that they created posts about these contents and shared them through the CMHC’s Facebook or Instagram accounts and workers said that this also allowed them to provide guidance about seeking mental health care in the CMHCs. Indeed, some users and relatives commented that they learned about the service and sought help due to these social media posts.

### 3.3 Difficulties in the provision of mental health care through telehealth

#### 3.3.1 Lack of technological resources

Most directors, workers of all CMHCs agreed that there was a lack of computers, cameras, printers, and cell phones. In addition, they said that the internet connection at the CMHCs was “unstable”, “of poor quality”, and sometimes absent for hours or even days. Local policymakers argued that CMHCs were provided with some technological resources in the pandemic, but national policymakers recognized these were “insufficient”. In this scenario, workers said that they had to use their own equipment, such as laptops and cell phones, or their own internet data.

Likewise, most local and national policymakers, CMHCs' workers and directors commented that the national guidelines required workers to register their performed activities virtually, even though CMHCs did not have telehealth platforms, electronic medical records, or electronic prescriptions.

Similarly, users and their relatives also identified some difficulties in terms of technology. For example, some users mentioned that it was difficult to receive remote care because they lacked internet in their houses.

*I didn’t have a lot of internet data … or I didn’t have a lot of megabytes, so my internet ran out, and I couldn’t receive the appointment (New user, CMHC3).*

#### 3.3.2 Saturation of communication channels

Some CMHCs' workers and directors reported that, at the beginning of the pandemic, several users suspended their treatments, they suggested that this occurred due to fear of moving or because they did not know if the CMHC continued working. As a result, health workers said that they reached the users by phone or social media (e.g. Facebook and Instagram), to ask about their health and inform that they continued providing care at the CMHC.

Nonetheless, once the cases of COVID-19 decreased, CMHCs' workers said that they noticed a higher demand of adult users, but the number of workers remained scarce. According to these workers, some possible explanations for this increase were that users were allowed to receive care in any CMHC, regardless of their place of residence; and because the MoH created a hotline (*Linea 113*) in which any person could call to request medical guidance, including information about the nearest CMHC to receive mental health care. For these workers, this resulted in more people reaching the CMHCs and insufficient staff for the new number of users. Moreover, administrative workers of two CMHCs reported having worked with fewer staff because many workers requested medical leaves due to COVID-19 infections. Policymakers and directors explained that they had a shortage of workers in CMHCs due to worker resignations or because the staff requested medical leave after being infected with COVID-19, giving them two weeks for their recovery by regulation, while the other workers had to take over the functions of those who were infected. Local policymakers mentioned that one of the measures they had to take, based on the high demand, was to extend the appointment schedule, so in some CMHCs, patients had to wait three months to receive care.

*… as a result of the pandemic, through the MoH hotline, all users were recommended to attend here. So, the number of users increased, right? And we had a lack of doctors because we only had 2 psychiatrists (Administrative worker, CMHC4).*

On the other hand, users explained that they telephoned the CMHC to schedule their appointments, but they noticed that administrative workers did not have enough time to schedule the appointments on time and their slots were usually full. Therefore, users expressed that they had to wait several days or weeks to be able to schedule an in-person or virtual appointment.

*Sometimes, when someone wants to schedule an appointment by phone, the appointments run out quickly … only one CMHC phone number answered, so I had to call again to make an appointment (New user, CMHC4).*

#### 3.3.3 Care modality preference

Even though the use of telehealth brought some benefits to the CMHCs' providers and users, most participants said that they preferred in-person care. For instance, health and administrative workers stated that telehealth represented a “double job” because they did not have a telehealth platform, and they had to record their activities both virtually and in paper. Likewise, users and their relatives considered that the in-person treatment was better. They perceived that the provision of care through phone calls or video calls limited the quality of care, indicating that they could not “feel the presence” of the workers or that connectivity issues made it hard to communicate.

*On the phone, I felt that … I don’t know, their presence was not there (New user, CMHC2).*

Yet, users commented that they recognized that having to travel to the CMHC, compared with having a call, was a disadvantage for them since it meant spending money and time. Despite local and national policymakers noted the complications reported by users and workers, they proposed expanding telehealth in the future, but improving the technological infrastructure to make this possible.

*I believe that the pandemic is an opportunity to move towards telehealth. These work modalities began in the pandemic, and we have seen that telehealth is convenient for some users, mainly to reduce their risk of (getting COVID-19 because of) attending (the CMHC), (and because) it is less expensive. If people are stable, they can make their appointments remotely. I think that telehealth is important and should be maintained because it can save us money in some aspects, such as (investing in) macro-regional training (National policymaker).*

## 4 Discussion

### 4.1 Main findings and interpretation

This qualitative study aimed to describe and analyze the perceptions and experiences of different key stakeholders (i.e. CHMCs’ users and their relatives, health and administrative workers, as well as local and national policymakers) to understand the use of telehealth in CMHCs during the COVID-19 pandemic in Lima and Callao, Peru. Our main finding is that all participants agreed that telehealth allowed them to continue delivering mental health care in the CMHCs, an important statement considering that many Latin American countries stopped their provision of mental health services at the beginning of the pandemic (Tausch et al., 2021).

Although the Peruvian MoH established various regulations aimed to guarantee the provision of care in different mental health services (Peruvian Ministry of Health, 2020d), including the CMHCs (Peruvian Ministry of Health, 2020b), only some of these policies were known by the CMHCs staff. For example, the participants mentioned policies to prioritize the in-person care provided by doctors, nurses, and administrative staff, as well as a policy to increase the use of technological tools to provide remote care for the other professionals. However, there were other relevant regulations related to telehealth that were not mentioned by the interviewed workers, such as the creation of the electronic medical record and the training of workers in the use of digital tools, which may reflect that not all policies are communicated with workers, remaining only on paper (Peruvian Ministry of Health, 2020c). In order to assure these policies' adoption, both local and national policymakers should guarantee their dissemination to all the stakeholders involved, especially among those who have to implement the policies within the health services (Tausch et al., 2021). On the other hand, policies were created for CMHCs to use telehealth as a result of the pandemic (Peruvian Ministry of Health, 2020c) and many workers and directors mentioned that, at the beginning of the pandemic, the Health Directorates informed them that they should migrate to telehealth. However, this policy could not be carried out due to a lack of technological and logistical resources. It is therefore necessary to design policies based on the capabilities of CMHCs. Despite these regulations, the CHMCs did not have sufficient capacity or resources, so the Health Directorates gave them freedom to make adjustments in the implementation of the policies (Peruvian Ministry of Health, 2020b).

The study participants recognized various benefits of using telehealth to provide mental health care. Health workers emphasized that telehealth allowed access to users who were physically far from the facilities. This was especially important during the first phase of the pandemic, when many people moved from Lima to their hometowns, far from the country capital, where they could find the support of their families (Fort et al., 2021). The possibility to receive remote care allowed them to continue their treatments. For example, more relatives were able to easily attend family therapy or family education sessions since the use of telehealth (Appleton et al., 2021). Previous studies have found that adoption rates for tele mental health treatment were high, ranging from 48% to 100% (Anton et al., 2021; Miu et al., 2021). Similarly, some users and their relatives felt relieved that they did not have to attend the CMHC to receive care due to fear of being infected. As other studies reported, people with mental disorders were one of the populations that experienced the greatest fear of being infected by COVID-19 (Quadros et al., 2021), so the use of telehealth helped them to reduce this concern. Finally, another benefit mentioned by health workers was that the use of telehealth allowed them to follow up and confirm appointments through phone calls. This is aligned to other studies that have found that telehealth contributes to perform more frequent follow-ups and consultations, and to have better treatment adherence because “users only have to answer a call” (Gomez et al., 2021). Even in HICs, calls were the most widely used and preferred modality by users (Appleton et al., 2021).

Despite these benefits, users and workers argued that they preferred in-person to remote care. Although some systematic reviews have shown that psychotherapy is equally effective remotely and in person (Drago et al., 2016), some qualitative studies have found that workers and users negatively rate the impact of remote consultations on their therapeutic relationship (Frye et al., 2022), and they are less likely to feel confident talking about sensitive topics (Galvin et al., 2022). For example, women who experienced domestic violence, have also reported difficulties accessing a private space for telehealth in LMICs (Munthali-Mulemba et al., 2022). If these woman were close to their abusive partners, women felt that they could be heard, so they decided to suspend or postpone their sessions, affecting the treatment (Munthali-Mulemba et al., 2022). Other studies found that doctors perceived that the use of telehealth generated a loss of personal connection, especially to observe the users' personal language and to establish a close bond that generates confidence to talk about personal issues (Gomez et al., 2021).

This preference for in-person care could be also related to the difficulties that CMHCs' users and workers experienced due to the rapid change from face-to-face to remote care, such as the lack of electronic devices, inadequate internet, a perception of increased workload, lack of training to adapt virtual care to telehealth or how to use digital tools (e.g. Zoom, Google Drive) and a lack of electronic medical records. These difficulties were also found in studies performed in LMICs, which workers and patients reported lack of access to internet and electricity, limiting their access to mental health services during the pandemic (Kola et al., 2021). In LMICs, it is crucial to recognize the influence of the social determinants of health on the delivery of health services. For example, individuals with severe mental health conditions may also face socio-economic challenges, which can hinder their ability to prioritize digital connectivity and obtain the necessary devices for ongoing telecare (Appleton et al., 2021). Besides, older patients may encounter financial limitations and encounter additional difficulties in utilizing technology, consequently, phone communication becomes their primary mode of interaction. These patients have expressed a strong preference for returning to in-person consultations after the pandemic (Li et al., 2022), which is consistent with our findings.

However, policymakers were enthusiastic with the provision of remote care using telehealth and the Peruvian MoH is promoting the implementation of electronic medical records nationwide, a system that includes specific modules for mental health care at the CMHCs (Peruvian Ministry of Health, 2017). Based on our findings, public mental health services will need a better technological infrastructure and an efficient internet connectivity to ensure a smooth implementation of electronic medical records, such as in countries that successfully provide remote or hybrid care (Carlbring et al., 2017). Lastly, although two technological platforms (i.e. MentalCom and e-QHALI), similar to an electronic medical record were used by some CMHCs, workers said that these platforms were only used by administrative workers to schedule appointments. However, health workers did not use them due to a lack of desktops and printers. Future initiatives to provide remote care should ensure that all the functions of the platforms can be used by both health and administrative workers, providing them with the technological infrastructure. This is consistent with previous studies that reported a lack of initial information, training to use telehealth platforms, and measures to assure privacy (Li et al., 2022). Besides, users and health workers reported experiencing distress due to not knowing how to use the technology (Li et al., 2022).

### 4.2 Public health implications

Similar to our findings, a scoping review showed that health workers and users from LMICs prefer to use free and easy to use tools (i.e. Google Drive and Google Meet) due to a lack of technological infrastructure, but these tools do not ensure the privacy of user data (Mahmoud et al., 2022). Although cloud storage has enabled a greater ease of access to files across devices and reduced the risk of theft and loss of paper records, storing information online also require providers' knowledge of encryption standards, password and device management and maintenance practices (Su et al., 2020). Electronic medical records placed in cloud storage increase the risk of inadvertent confidentiality breaches and unauthorized remote access, including populations with low socioeconomic status or located in rural areas with less technological protections, which could further put their data stored in the cloud at risk, so it is important to set clear policies for data security and protocols for electronic data exchange (Su et al., 2020). According to a Peruvian law (Information Technology General Office, 2020) and the global requirement of digital health (WHO, 2021), health data requires a high level of security and protection, thus our study points to the need to generate surveillance strategies on this legal basis to guarantee the confidentiality of information in CMHCs.

Our results provide valuable information that policymakers should consider when implementing telehealth services in CMHCs. For instance, although using telehealth to provide mental health services seems to be cost-effective (Sunjaya et al., 2020), the Government has to ensure internet connectivity for its use and the provision of devices, such as desktops and printers. Other potential barriers must be considered, such as the low acceptance of workers who perceive that the implementation of a new system will generate double work. Research on this regard suggests that the implementation of a system of medical records, such as SIHCE in Peruvian primary care settings, should respond to a clearly perceived need by workers and involve them as partners in the implementation process (Pan American Health Organization, 2016).

Finally, an important finding in our study was that CMHCs workers indicated that they had no one to turn to when they experienced mental health problems. Indeed, one regulation established that all health personnel should receive psychological support and this support was led by the CMHCs (Peruvian Ministry of Health, 2020d); but there was not any guidance on providing psychosocial care to the CMHCs workers when they were the ones experiencing mental health issues. Thereby, there is a need to guarantee the access to psychosocial support for mental health workers and to adapt these measures to the context of providing remote care, since they are a vulnerable population in health emergencies, such as COVID-19 (Kola et al., 2021).

### 4.3 Strengths and limitations

One strength of this study is the diversity of stakeholders involved, which ensures a variety of complementary perspectives and experiences that also reinforce the internal validity of the study. One limitation is that interviews were conducted between September 2021 and March 2022, between a year and a half and two years since the start of the pandemic, which could have led to recall biases of some of the participants' experiences. However, this timeframe also allowed participants to reflect about their successes, challenges, and lessons learned throughout the pandemic, and share with us their reflections and insights.

## 5 Conclusion

We found that the CMHCs in Lima and Callao rapidly moved to the provision of remote care during the pandemic, following national policies to reduce in-person care in public health services. There was consensus among participants that the use of telehealth allowed the continuation of mental care, even for those users who moved to other cities, and helped to reduce users' and relatives' fear of getting COVID-19. Despite these benefits, most workers, users and relatives deemed that the provision of mental health care in person is more effective. This perception might be related to the difficulties found to use telehealth in the CMHCs, such as a lack of devices, poor internet connection, and the perception of increased workload. Considering that the Peruvian Government is promoting the implementation of electronic medical records, it would be important to learn from the CMHCs experiences during the pandemic to assure a better use of technology in public mental health services.

## Funding

This work was supported by the Foreign, Commonwealth & Development Office (FCDO), the Medical Research Council (MRC) and Wellcome [grant number MR/V014919/1].

## Ethics approval and consent to participate

Approval was obtained from the Universidad Peruana Cayetano Heredia’s Ethics Committee in Peru (Approval record 573-33-20). The procedures used in this study adhere to the tenets of the Declaration of Helsinki. Informed consent was obtained from all participants included in the study.

## Consent for publication

Not applicable.

## CRediT authorship contribution statement

**Rubí Paredes-Angeles:** Conceptualization, Validation, Formal analysis, Investigation, Writing – original draft, Approval of the final version. **Victoria Cavero:** Methodology, Formal analysis, Investigation, Writing – review & editing, Approval of the final version. **Ana Lucía Vilela-Estrada:** Conceptualization, Formal analysis, Investigation, Writing – review & editing, Approval of the final version. **Noelia Cusihuaman-Lope:** Conceptualization, Formal analysis, Investigation, Writing – review & editing, Approval of the final version. **David Villarreal-Zegarra:** Investigation, Writing – review & editing, Approval of the final version. **Francisco Diez-Canseco:** Methodology, Investigation, Resources, Writing – review & editing, Supervision, Approval of the final version.

## Declaration of competing interest

The authors declare that they have no known competing financial interests or personal relationships that could have appeared to influence the work reported in this paper.
